# Awareness Among US Adults of Dental Sealants for Caries Prevention

**DOI:** 10.5888/pcd16.180398

**Published:** 2019-03-14

**Authors:** Michele L. Junger, Susan O. Griffin, Srdjan Lesaja, Lorena Espinoza

**Affiliations:** 1 Centers for Disease Control and Prevention, Division of Oral Health, Atlanta, Georgia; 2 DB Consulting Group, Atlanta, Georgia

## Abstract

**Introduction:**

Dental sealants applied in childhood can help prevent caries, but knowledge of the availability of sealants and their function is not widespread. We assessed knowledge of dental sealants among US adults and adult parents of children younger than 18 and the differences in knowledge among demographic and socioeconomic groups.

**Methods:**

We used data on 3,550 respondents to the 2015 FallStyles B survey of noninstitutionalized US adults aged 18 or older. Authors constructed estimates by using weights provided to reflect the distribution of the US population. Knowledge of dental sealants was assessed by sex, age, race/ethnicity, education, household income, and parental status. Multivariate analysis was conducted by using a main effects logistic regression model.

**Results:**

Overall, 46.3% of adults and 55.1% of parents of children younger than 18 had knowledge of dental sealants. Sealant knowledge was highest among parents, women, respondents aged 45 to 59, and respondents with incomes greater than 200% of the federal poverty level and more than a high school education. Non-Hispanic blacks had less than half the odds of non-Hispanic whites of having knowledge of sealants (adjusted odds ratio [OR] = 0.4), and nonparents had half the odds as parents (OR = 0.5) of knowing. The strongest predictors of parental sealant knowledge were race/ethnicity, sex, and income.

**Conclusion:**

Disparities in sealant knowledge correspond to disparities in sealant prevalence. Increasing knowledge among low-income and racial/ethnic minority parents could reduce disparities in sealant prevalence and untreated caries.

**MEDSCAPE CME**

Medscape, LLC is pleased to provide online continuing medical education (CME) for this journal article, allowing clinicians the opportunity to earn CME credit.

In support of improving patient care, this activity has been planned and implemented by Medscape, LLC and *Preventing Chronic Disease*. Medscape, LLC is jointly accredited by the Accreditation Council for Continuing Medical Education (ACCME), the Accreditation Council for Pharmacy Education (ACPE), and the American Nurses Credentialing Center (ANCC), to provide continuing education for the healthcare team.

Medscape, LLC designates this Journal-based CME activity for a maximum of 1.00 *AMA PRA Category 1 Credit(s)™*. Physicians should claim only the credit commensurate with the extent of their participation in the activity.

All other clinicians completing this activity will be issued a certificate of participation. To participate in this journal CME activity: (1) review the learning objectives and author disclosures; (2) study the education content; (3) take the post-test with a 75% minimum passing score and complete the evaluation at http://www.medscape.org/journal/pcd; (4) view/print certificate.

Release date: March 14, 2019; Expiration date: March 14, 2020

**Learning Objectives**

Upon completion of this activity, participants will be able to:

1. Assess knowledge among US adults of dental sealants, based on an analysis of data from the 2015 Styles Survey
2. Determine factors associated with knowledge among US adults of dental sealants, based on an analysis of data from the 2015 Styles Survey
3. Evaluate the clinical implications regarding knowledge among US adults of dental sealants, based on an analysis of data from the 2015 Styles Survey

**EDITOR**

Rosemarie Perrin

Editor, *Preventing Chronic Disease*

Disclosure Rosemarie Perrin has disclosed no relevant financial relationships.

**CME AUTHOR**

Laurie Barclay, MD

Freelance writer and reviewer

Medscape, LLC

Disclosure: Laurie Barclay, MD, has disclosed no relevant financial relationships.

**AUTHORS**

Michele Junger, DDS, MPH

Centers for Disease Control and Prevention

Division of Oral Health

Atlanta, GA

Disclosure: Michele Junger, DDS, MPH, has disclosed no relevant financial relationships.

Susan O. Griffin, PhD

Centers for Disease Control and Prevention

Division of Oral Health

Atlanta, GA

Disclosure: Susan O. Griffin, PhD, has disclosed no relevant financial relationships.

Srdjan Lesaja, MS

DB Consulting, Inc

Division of Oral Health

Atlanta, GA

Disclosure: Srdjan Lesaja, MS, has disclosed no relevant financial relationships.

Lorena Espinoza, DDS, MPH

Centers for Disease Control and Prevention

Division of Oral Health

Atlanta, GA

Disclosure: Lorena Espinoza, DDS, MPH, has disclosed no relevant financial relationships.

**Summary**

**What is already known about this topic?**

Dental sealants are an effective way to prevent cavities. However, sealants are underused, especially in those children who are at the highest risk for untreated cavities.

**What is added by this report?**

In 2015, about half of adults knew the purpose of dental sealants. Parents had more knowledge than the overall population. We assessed the differences in knowledge among demographic and socioeconomic groups.

**What are the implications for public health practice?**

By understanding disparities in sealant knowledge, dental professional and public health organizations can develop targeted oral health promotion and education programs. Reaching low-income and racial/ethnic minority parents and parents with only young children could reduce disparities in sealant knowledge and untreated cavities.

## Introduction

Although largely preventable, dental caries is one of the most common chronic diseases among children and adolescents (1). National data from 2011 through 2014 show that approximately 18% of children aged 6 to 11 and 58% of children aged 12 to 19 in the United States had treated or untreated caries in their permanent teeth (2). Disparities in caries prevalence exist across races and ethnicities and family income levels, and prevalence is highest among minority and socio-economically disadvantaged populations (2). If left untreated, dental caries can cause pain, speech problems, and missed time from school (1).

About 90% of caries in permanent teeth occur in the posterior teeth (3). Dental sealants are widely recommended by professional health organizations (4,5) because they prevent about 90% of posterior caries one year after placement and about 50% 5 years after placement (6). Prevalence of sealant use in children aged 6 to 11 rose 12.4 percentage points from 1999–2004 to 2011–2014, from 31.1% to 43.6% (7). Children from low-income households (<185% of the Federal Poverty level [FPL), however, are about 20% less likely to receive a sealant than children from higher income households (7).

Recent attention to health literacy highlights the complex relationship between knowledge and actions that support health (8). A recent analysis of sealant prevalence in children found that among high income parents (≥100% of the federal poverty level), sealant prevalence increased with parental education (a proxy for health literacy) (9). Because oral health literacy is required to make informed health decisions and can affect receipt of services, determining public knowledge of the purpose of sealants is important. No national data characterize knowledge of sealants among all US adults and among parents of children younger than 18. We assessed knowledge of the purpose of sealants and the differences in knowledge among demographic and socioeconomic groups. Information from our study provides a baseline for future studies of sealant knowledge and can be used to identify need for promoting oral health education and increasing oral health literacy.

## Methods

Styles is a consumer survey of US adults that is conducted in multiple waves throughout the year. Data for our study were taken from the FallStyles B 2015 survey, which was conducted from September 28 through October 16, 2015 (unpublished raw data from Porter Novelli Public Services *Styles 2015 Survey* via Deanne Weber). The 2015 FallStyles B survey is a follow-up to the SpringStyles survey. FallStyles was obtained from 3 sampling waves of GfK KnowledgePanel (GfK), a probability-based online panel of 55,000 noninstitutionalized adults that is representative of the adult US population (10). The first wave of Styles, SpringStyles, was sent to a random sample of panelists aged 18 or older (n = 11,028). SpringStyles included questions about general media habits, product use, interests, and lifestyle. The second wave, SummerStyles, which included questions on health orientations and practice, was sent to a random sample of respondents to the SpringStyles survey (n = 6,172). FallStyles, the third wave, was released in 2 separate surveys — A and B. FallStyles B, which included our question, “Which of the following best describes the purpose of dental sealants?,” was sent to 4,665 respondents who completed the SummerStyles survey and had 3,550 respondents, a response rate of 76.1%. FallStyles data were weighted by sex, age, household income, race/ethnicity, household size, education, census region, metro status (if respondents live in a metropolitan area or not), and prior internet access to create a sample reflective of the US Current Population Survey proportions. We were granted access to the FallStyles B data through a data-use agreement with Porter Novelli Public Services. Our study was exempt from institutional review board review because personal identifiers were not included.

Dental sealant knowledge, our dependent variable, was recorded as present if a respondent selected “to prevent tooth decay” in answer to the multiple choice question, “Which of the following best describes the purpose of dental sealants?” Incorrect responses included “to fill cavities,” “to improve appearance of teeth,” “to hold dentures in place,” “to protect teeth while playing sports,” or “I don’t know.” Independent variables included sex (male, female), age (18–29, 30–44, 45–59, ≥60 y), race/ethnicity (non-Hispanic white, non-Hispanic black, Hispanic, other), education (<high school diploma, high school diploma, more than a high school diploma), parental status (having a child aged <18 y), having young children (oldest child aged <6 y), and household income relative to the FPL (based on income and household size). Household poverty status was calculated by using self-reported household size and family income and applying the Department of Health and Human Services 2015 US Federal Poverty Guidelines (11). Household income was dichotomized into 2 categories: poor (≤200% of the FPG) and not poor (>200% of the FPG).

We examined the distribution of independent variables for all adults and for parents. Because sealants typically are delivered to children and adolescents under parental authority, all analyses were conducted both for all adults and for parents. We used the χ^2^ test of independence to determine if sealant knowledge was associated with our explanatory variables. To explore factors associated with sealant knowledge after controlling for covariates, we performed multivariate analyses by using a main effects logistic regression model. Adjusted odds ratios and 95% confidence intervals were calculated for overall adult and parental knowledge. All reported findings were significant at *P* < .05.

We used SAS version 9.3 (SAS Institute Inc) for all data management, analysis, and modeling. Missing data represented a small percentage of the overall sample: 7 respondents, 2 of whom were parents, did not respond to the sealant question. Because we did not assume that failure to respond meant that a respondent did not know the answer to the question, missing data were excluded.

## Results

Of the 3,550 adult respondents to the 2015 FallStyles B survey, 27.5% indicated that they had at least one child younger than 18. Most of both the overall adult and parent samples were non-Hispanic white, were not poor, and had more than a high school education. Approximately half of the adult sample was aged 30 to 59, compared with over 80% of the parent sample (Table 1).

**Table 1:** Demographic Characteristics of Respondents (N = 3,550), Awareness Among US Adults of Dental Sealants for Caries Prevention, FallStylesB Survey^a^, 2015

Characteristic	All Adults % (SD), N = 3,550	Parents^b^, % (SD), N = 716
**Sex**		
Male	48.7 (65.5)	48.2 (58.9)
Female	51.3 (65.5)	51.8 (58.9)
**Age, y**		
18–29	21.3 (59.6)	14.3 (45.5)
30–44	25.0 (59.6)	56.0 (58.9)
45–59	26.3 (53.6)	26.2 (48.2)
≥60	27.4 (53.6)	3.6 (21.4)
**Race/ethnicity**		
Non-Hispanic white	66.3 (65.5)	58.8 (58.9)
Non-Hispanic black	11.3 (41.7)	11.3 (37.5)
Hispanic	14.8 (47.7)	21.2 (50.8)
Other	7.6 (41.7)	8.7 (40.1)
**Education**		
<High school diploma	11.6 (47.7)	13.6 (50.8)
High school diploma	29.7 (59.6)	21.6 (45.5)
>High school diploma	58.7 (65.5)	64.8 (58.9)
**Household income, % of federal poverty level**		
≤200	29.8 (59.6)	31.5 (56.2)
>200	70.2 (59.6)	68.5 (56.2)
**Parent^b^**		
Yes	27.5 (59.6)	—
No	72.5 (59.6)	—
**Sealant knowledge**		
Yes	46.3 (65.6)	55.1 (58.9)
No	53.7 (65.5)	44.9 (58.9)

Abbreviations: —, not applicable; SD, standard deviation.

a FallStyles B, a product of GfK’s KnowledgePanel (10) was sent to 4,665 participants and had 3,550 respondents, a response rate of 76.1%.

b Of a child aged <18.

Dental sealant knowledge among all adults was 46.3% (Table 2). In the bivariate analysis, sealant knowledge was associated with all independent variables. More than half of respondents who were parents, who had more than a high school education, or who were women, aged 45 to 59 years, non-Hispanic white, and not poor had sealant knowledge. In the multivariate analysis, all independent variables were significant. Adults who were non-Hispanic black, poor, had not graduated from high school, or were not parents had half the odds of knowing of dental sealants as those who were non-Hispanic white, not poor, had more than a high school education, or were parents.

**Table 2:** Knowledge of Dental Sealants Among Respondents (N = 3,550), Awareness Among US Adults of Dental Sealants for Caries Prevention, FallStylesB^a^ Survey, 2015

Characteristic	% (SD), N = 3,550	*P* Value	AOR (95% CI), N = 3,550	*P* Value
**Overall**	46.3 (65.5)	—	—	—
**Sex**				
Male	42.1 (89.4)	<.001	0.7 (0.6–0.8)	<.001
Female	50.2 (89.4)	1 [Reference]		
**Age, y**				
18–29	40.6 (154.9)	.003	0.7 (0.5–0.9)	<.001
30–44	45.3 (137.0)		0.6 (0.5–0.8)	
45–59	52.0 (113.2)	1 [Reference]		
≥60	46.1 (107.2)	003	0.9 (0.7–1.1)	<.001
**Race/ethnicity**				
Non-Hispanic white	52.1 (71.5)	1 [Reference]		
Non-Hispanic black	29.0 (184.7)	<.001	0.4 (0.3–0.5)	<.001
Hispanic	38.8 (178.7)		0.7 (0.6–0.8)	
Other	35.6 (274.1)		0.5 (0.4–0.7)	
**Education**				
<High school diploma	28.2 (214.5)	<.001	0.5 (0.4–0.6)	<.001
High school diploma	44.0 (113.2)		0.8 (0.7–1.0)	
>High school diploma	51.0 (77.5)	1 [Reference]		
**Household income, % of federal poverty level**				
≤200	31.8 (113.2)	<.001	0.5 (0.4–0.6)	<.001
>200	52.4 (71.5)	1 [Reference]		
**Parent^b^**				
Yes	55.1 (131.1)	1 [Reference]		
No	42.9 (71.5)	<.001	0.5 (0.4–0.6)	<.001

Abbreviations: —, not applicable; AOR, adjusted odds ratio; CI, confidence interval; SD, standard deviation.

a FallStyles B, a product of GfK’s KnowledgePanel (10) was sent to 4,665 participants and had 3,550 respondents, a response rate of 76.1%.

b Of a child <18.

Approximately 55% of parents had knowledge of dental sealants. Knowledge among parents was consistently higher compared to knowledge among all adults across all subcategories (Table 2). The greatest difference between adult and parental sealant knowledge was among respondents aged 60 or older. In the bivariate analysis, parental sealant knowledge varied significantly among all variables except age. Parental knowledge mirrored that of adults in general: higher among respondents who were women, non-Hispanic white, had higher-incomes, had more than a high school education, or had older children. Parents who were male, members of racial/ethnic minority populations, poor, less educated, or had children aged under 6 all had less knowledge. (Table 3) In the multivariate analysis, sex, race/ethnicity, and income remained significantly associated with parental knowledge.

**Table 3:** Knowledge of Dental Sealants among US Parents of a Child Aged <18, Awareness Among US Adults of Dental Sealants for Caries Prevention, FallStylesB^a^ Survey, 2015

Characteristic	% (SD, N = 716	*P* Value	AOR (95% CI), N = 716	*P* Value
**Overall**	55.1 (58.9)	—	—	—
**Sex**				
Male	48.2 (83.0)	.003	0.5 (0.4–0.7)	<.001
Female	61.6 (83.0)	1 [Reference]		
**Age, y**				
18–29	42.6 (176.6)	.06	0.6 (0.4–1.2)	.31
30–44	55.0 (77.6)		0.8 (0.6–1.3)	
45–59	60.6 (99.0)	1 [Reference]		
≥60	65.8 (264.9)	.06	1.7 (0.6–4.5)	.31
**Race/ethnicity**				
Non-Hispanic White	65.0 (66.9)	1 [Reference]		
Non-Hispanic Black	38.7 (179.3)	<.001	0.4 (0.2–0.6)	<.001
Hispanic	44.1 (141.8)		0.5 (0.3–0.7)	
Other	36.7 (232.8)		0.3 (0.2–0.6)	
**Education**				
<High school diploma	39.7 (200.7)	.04	0.8 (0.5–1.4)	.79
High school diploma	55.2 (120.4)		0.9 (0.6–1.4)	
>High school diploma	58.3 (66.9)	1 [Reference]		
**Household income, % **federal poverty level				
≤200	44.3 (109.7)	<.001	0.7 (0.5–1.0)	.03
>200	60.0 (66.9)	1 [Reference]		
**Age of oldest child, y**				
<6	49.2 (93.7)	.02	0.7 (0.5–1.0)	.05
≥6	59.9 (74.9)	1 [Reference]		

Abbreviations: —, not applicable; AOR, adjusted odds ratio; CI, confidence interval; SD, standard deviation.

a FallStyles B, a product of GfK’s KnowledgePanel (10) was sent to 4,665 participants and had 3,550 respondents, a response rate of 76.1%.

## Discussion

We found that approximately 50% of adults overall and 55% of parents knew the purpose of dental sealants. Results from our study are consistent with previous research that found adult sealant knowledge was higher among women, non-Hispanic whites, parents, and those with more than a high school education and who were not socioeconomically disadvantaged. (12–15). Additional variables found in the literature to be associated with adult knowledge were marital status, past-year dental visit, and being dentate (having teeth) (13–15). We did not include these variables in our analysis because FallStyles did not include questions on use of dental care or dentate status, and parental status was highly correlated with marital status. Consistent with findings from other studies (14,15), our analysis found that parental status was a strong predictor of sealant knowledge, and parental knowledge was associated with sex, race/ethnicity, and income (16,17).

A factor associated with increased parental sealant knowledge not included in our study is children’s use of dental care. A survey of Australian parents found that dentists were parents’ main source of dental information and that sealant awareness was associated with frequency of dental visits, type of dental center attended, and discussion of caries prevention with a dental professional (18). Similarly, studies have found the presence of sealants to be associated with a recommendation from a dental health care professional, having dental insurance and a regular source of dental care for the child, knowing of or being exposed to information about sealants, and sources of sealant information (19–22).

Studies indicate that children of parents with knowledge of dental sealants are more likely to have dental sealants (19,20). However, these studies did not assess whether knowledge was obtained before or after the child’s receipt of sealants, whether knowledge was a driving factor for the parent to seek the intervention, or if knowledge was a result of receiving the intervention. Nonetheless, these studies highlight the importance of sealant knowledge and suggest that discussion of sealants with a dental professional increases parental sealant knowledge and may lead to increased sealant prevalence.

Although knowledge without access to dental care will likely not change behavior, neither will access without knowledge (9). A basic premise of health literacy is that people must know about services to benefit from them. To make an informed decision whether to accept sealants in a clinical setting or to allow a child to participate in a school program, parents must have knowledge of dental sealants. Dental professionals play an important role in educating parents and caregivers about these programs and increasing sealant knowledge. Studies indicate that dentists can successfully persuade patients to accept procedures when the dentist has better knowledge than the patient about the needed procedure. This could explain why when states provide incentives for sealant placement for children covered by Medicaid, an incentive for dentists to increase sealant knowledge, more sealants are placed (23).

The American Academy of Pediatric Dentistry (AAPD) provides guidance on preventive dental services and anticipatory guidance for children (24). For children aged 2 to 6, AAPD recommends that dental health care personnel provide sealants for caries-susceptible primary molars and permanent molars, premolars, and anterior teeth; children should be reassessed at recall appointments to determine the need for new sealants or maintenance of existing sealants. In addition, the American Dental Association supports the use of sealants and encourages dentists to speak to their patients or parents about them (4). For parents of young children, especially those who are poor or from racial/ethnic minorities, initiating these discussions as early as possible could better prepare parents for sealant placement. However, because dental care is reduced among low-income and racial/ethnic minority families and among parents with only very young children, relying on dental professionals to provide sealant information is problematic (25).

School nurses and pediatricians could help increase knowledge of dental sealants. School sealant programs are a successful and cost-effective strategy to increase sealant receipt among children who typically lack access to clinical dental care (7,26). A major barrier to successful implementation of these programs is low consent rates, which might be influenced by parental lack of sealant knowledge. Our finding that parents with only children younger than 6 have less knowledge of sealants is consistent with a recent study in Maryland that conducted a focus group of low-income parents or caregivers of children aged 6 and younger and pregnant women. That study found that very few of the participating parents had heard of dental sealants (27).

Our study had limitations. Styles uses market research databases and is not intended for health surveillance. Survey respondents had already replied to 2 surveys before completing the FallStyles B survey and were more likely to be responders than the average population. Although our data cannot be considered nationally representative, a study comparing Styles to 9 items from the Behavioral Risk Factor Surveillance System found Styles data to be both reliable and valid (28). Styles was only offered in English and does not represent non-English speakers. In addition, compared with similar previous surveys, the 2015 survey included a markedly decreased number of parents of children younger than 18. Because the sample size of parents was small, parental knowledge results should be interpreted with caution. Next steps that might be helpful in developing health promotion and educational efforts at the national, state, or local level include identification of stakeholders and potential collaborators, collection of area-specific data and data among non-English speaking populations (especially non-English–speaking parents), and the identification of methods successfully used to improve knowledge among selected populations. Efforts should be made to standardize questions used to assess health literacy so results across different studies can be compared.

Although prevalence of dental sealants has increased, they are still underutilized among children at risk for untreated caries (7). We found corresponding disparities in knowledge of the preventive purpose of sealants. The dental community remains a major source of information on the preventive benefits of sealants. Further efforts by dental professional organizations and public health organizations to develop oral health promotion and education programs to reach low-income and racial/ethnic minority parents and parents with only young children could reduce disparities in sealant knowledge and untreated dental caries.

## Post-Test Information

To obtain credit, you should first read the journal article. After reading the article, you should be able to answer the following, related, multiple-choice questions. To complete the questions (with a minimum 75% passing score) and earn continuing medical education (CME) credit, please go to http://www.medscape.org/journal/pcd. Credit cannot be obtained for tests completed on paper, although you may use the worksheet below to keep a record of your answers.

You must be a registered user on http://www.medscape.org. If you are not registered on http://www.medscape.org, please click on the “Register” link on the right hand side of the website.

Only one answer is correct for each question. Once you successfully answer all post-test questions, you will be able to view and/or print your certificate. For questions regarding this activity, contact the accredited provider, CME@medscape.net. For technical assistance, contact CME@medscape.net. American Medical Association’s Physician’s Recognition Award (AMA PRA) credits are accepted in the US as evidence of participation in CME activities. For further information on this award, please go to https://www.ama-assn.org. The AMA has determined that physicians not licensed in the US who participate in this CME activity are eligible for *AMA PRA Category 1 Credits™*. Through agreements that the AMA has made with agencies in some countries, AMA PRA credit may be acceptable as evidence of participation in CME activities. If you are not licensed in the US, please complete the questions online, print the AMA PRA CME credit certificate, and present it to your national medical association for review.

## Post-Test Questions

### Study Title: Awareness Among US Adults of Dental Sealants for Caries Prevention

#### CME Questions

1. You are advising a large dental practice regarding improving uptake of dental sealant use among their patients. On the basis of the analysis of data from the 2015 Styles Survey by Junger and colleagues, which one of the following statements about knowledge among US adults of dental sealants is correct?
  - Dental sealant knowledge, that is, knowing that their purpose is to prevent tooth decay, was 26.3% among all adults
  - Dental sealant knowledge among parents was 35.1%
  - More than half of respondents who were parents, who had some college education, or who were non-Hispanic white women aged 45 to 59 years and who were not poor had sealant knowledge
  - The Styles Survey findings differ markedly from those of previous studies
2. According to the analysis of data from the 2015 Styles Survey by Junger and colleagues, which one of the following statements about factors associated with knowledge among US adults of dental sealants is correct?
  - Compared with non-Hispanic whites, non-Hispanic blacks had less than half the odds of having knowledge of sealants (odds ratio, 0.4)
  - Adults in lower- vs higher-income households had one quarter the odds of having sealant knowledge, as did nonparents vs parents (odds ratio, 0.25 for both)
  - The strongest predictor of parental sealant knowledge was educational level
  - The Styles Survey showed that past-year dental visit and having teeth strongly predicted parental sealant knowledge
3. On the basis of the analysis of data from the 2015 Styles Survey by Junger and colleagues, which one of the following statements about clinical implications regarding knowledge among US adults of dental sealants is correct?
  - The American Academy of Pediatric Dentistry recommends that children aged 2 to 6 years receive sealants only for caries-susceptible primary molars
  - Only dental professionals should provide sealant information to parents
  - School sealant programs are expensive and ineffective
  - Improving knowledge among low-income and racial/ethnic minority parents and those with young children could lower disparities in sealant prevalence and untreated caries
